# Factors Associated With Fecal Calprotectin Sample Collection Compliance: An IBD Center Quality Improvement Project

**DOI:** 10.1093/crocol/otac042

**Published:** 2022-12-03

**Authors:** David Fenton, Natalie K Choi, Nicole M Garcia, Emma C Dyer, Nathaniel A Cohen, David T Rubin

**Affiliations:** Pritzker School of Medicine, University of Chicago, Chicago, Illinois, USA; Inflammatory Bowel Disease Center, University of Chicago Medicine, Chicago, Illinois, USA; Inflammatory Bowel Disease Center, University of Chicago Medicine, Chicago, Illinois, USA; Inflammatory Bowel Disease Center, University of Chicago Medicine, Chicago, Illinois, USA; Inflammatory Bowel Disease Center, University of Chicago Medicine, Chicago, Illinois, USA; Inflammatory Bowel Disease Center, University of Chicago Medicine, Chicago, Illinois, USA

**Keywords:** fecal calprotectin, noninvasive, IBD management, compliance, test completion

## Abstract

**Background:**

Fecal calprotectin (Fcal) is a noninvasive, inexpensive biomarker of disease activity. However, patient compliance with this test is variable and incompletely described. We assessed compliance rates with Fcal tests and identified factors associated with noncompliance.

**Methods:**

A retrospective chart review of patients with inflammatory bowel disease (IBD) who had a Fcal test ordered through our center between August 2021 and December 2021 was conducted. Demographic, clinical, disease, and test-related information were recorded. Patients with incomplete Fcal orders were sent a survey to better understand their reasons for noncompliance. Simple statistical analysis and and multivariable logistic regression modeling were performed.

**Results:**

Of 303 patients, 165 (54.4%) had an order for Fcal. Of the Fcal tests ordered, 55 (33.3%) were not completed. Remission of IBD, no prior Fcal completion, and tests ordered at a distant site were all associated with test noncompletion. A multivariable logistic regression revealed that history of a prior completed Fcal test is associated with subsequent test completion (odds ratio = 2.1, 95% confidence interval 1.9–35.5, *P* = .004). Patients who did not complete the test described the pandemic and third-party testing center issues as the most common reasons for noncompliance.

**Conclusions:**

In this single center experience with Fcal testing in patients with IBD, we identified that a history of incomplete Fcal testing and distant location of lab testing were significantly associated with noncompletion of the test. We provide practical guidance for future utilization and compliance, including the impact of home-based testing.

## Introduction

Inflammatory bowel diseases (IBDs), comprised of Crohn’s disease and ulcerative colitis, are chronic and debilitating conditions that greatly affect the health-related quality of life for patients.^1,2^ Unregulated intestinal inflammation can cause irreversible bowel damage and result in disease-related complications such as fistulae and strictures, abscesses, and cancer, often leading to subsequent hospitalizations and surgeries.^3–6^ Thus, proactive monitoring is an important method to measure disease activity and progression. At present, management of IBD is based on the “Treat-to-Target” methodology, which involves tightly monitoring gastrointestinal inflammation through the use of validated outcome measurements for inflammation and adjusting therapy accordingly.^7–9^ Although the clinical gold standard for assessing disease activity remains endoscopy due to the opportunity macroscopically and microscopically to evaluate inflammation, recent studies have shown that repeated endoscopic surveillance exposes patients to invasive procedures with the nonnegligible associated risks, is time consuming, and is poorly tolerated.^10–14^ As a result, the usage of clinical surrogate markers has become crucial not only to distinguish between active and inactive IBD-related disease, but also may play a role in stratifying patients at risk for exacerbation or those eligible for therapeutic de-escalation.^1^

Fecal calprotectin (Fcal) testing, a noninvasive surrogate tool used as part of IBD diagnostic algorithms, is integral to monitoring disease activity and response to therapy and also predicts clinical relapse.^15–19^ In Fcal testing, patient stool samples are collected to assess the protein biomarker levels, to determine neutrophilic infiltration within the intestinal mucosa, and to evaluate overall gut inflammation.^19,20^ In addition to being significantly correlated with endoscopic and histologic inflammation, Fcal assessment also presents as an inexpensive, effective diagnostic tool that circumvents many of patient-related difficulties associated with endoscopic procedures.^1,20^ Moreover, several randomized controlled trials evaluating the efficacy of Fcal have demonstrated that biochemical monitoring predicts disease outcomes and onset of relapses, ultimately allowing for expedited and evidence-guided therapeutic modifications.^21–23^ However, regular monitoring in chronic diseases such as IBD faces unique obstacles associated with patient compliance, and while Fcal has shown notable potential, factors related to compliance in test order fulfillment have largely remained unstudied.^24–27^

The objective of this study is to determine predictors that affect Fcal testing compliance, qualitatively understand patient perspectives for incompletion, and provide applicable recommendations based on these findings. We hypothesize that patients who were seen virtually, from out of state, or those who had labs ordered at third-party locations will be less likely to complete Fcal testing and have increased risk for testing noncompliance.

## Methods

### Study Design

A retrospective chart review of patient medical records from a single practitioner was conducted at the University of Chicago IBD Center between August 2021 and January 2022. The University of Chicago IBD Center supports several clinic locations across the [Institution City]: (1) Clinic Location 1 is located within the main University of Chicago campus on the Southern part of the city, and (2) Clinic Location 2 is off-site, located in the downtown area of the city. Patients were included if they had a prior Fcal test ordered during the study period. Patients were excluded if the Fcal test was ordered through an external institution, or if the Fcal order was still active within 2 weeks of a clinic visit (not expired and requiring a new order). This study was IRB exempt by the University of Chicago Institutional Review Board and approved as a quality improvement project by the Healthcare Delivery Science and Innovation Board (entry #752).

### Medical Information and Outcome Variables

Medical information reviewed included demographic information (age, sex, race/ethnicity, insurance type, and median income approximated by zip code), IBD-related medical history (diagnosis or disease phenotype), disease activity (determined from Harvey–Bradshaw Index [HBI] or Simple Clinical Colitis Activity Index [SCCAI] routinely collected during clinic visits), history of advanced therapies (small molecule or biological therapies), other comorbidities (hypertension, diabetes mellitus, etc.), and history of surgeries or hospitalizations. Patients with a HBI score less thanless than 5 or a SCCAI score less than or equal to 2 were considered to be in clinical remission. Relevant clinical encounter characteristics were also reviewed (clinic location, in-person vs telehealth clinic visit, patient location, and testing location). Patients seen in-person received a stool kit (comprised of necessary contents for sample collection and an instruction manual) when a Fcal test was ordered, unless a test was ordered through a third-party laboratory.

Status of Fcal completion was reviewed as our primary outcome variable. For patients with completed orders, we recorded the difference in days between the initial Fcal order and the date the Fcal order was completed by the patient (period to test completion).

All data were stored electronically in a REDCap^R^ database (see Supplementary Material 1 for the full list of recorded variables).^28,29^

### Prospective Survey

A brief prospective survey was developed to understand patients’ perspective of noncompliance. The survey asked basic understanding of what a Fcal test is, if they were notified the test was ordered, and whether they were made aware of its importance. If patients reported not knowing what the Fcal test consisted of, a brief description of the test was provided for education. Finally, respondents could select all reasons that may have affected whether they completed the test or enter a short response (see Supplementary Material 2 for the full questionnaire). All patients with incomplete Fcal tests were surveyed from June 2022 to July 2022.

### Statistical and Thematic Analyses

Statistical analysis was conducted using SPSS (IBM corporation, version 28) and R (Foundation for Statistical Computing, Vienna, Austria).^30,31^ Parametric data were analyzed using Student’s *t*-tests and Pearson chi-square tests where appropriate for continuous and categorical variables, respectively. Nonparametric data were compared using Mann–Whitney *U*-tests. A multivariable logistic regression and a multivariable linear regression were performed to determine significant predictors of Fcal compliance and longer periods to Fcal test completion, respectively. Predictor variables for the regressions included age, gender, and significant variables from descriptive statistics (IBD remission, clinic visit type, history of Fcal order completion, patient location, and testing location; *P* < .05). Significance for all analyses was determined at the threshold *α* = 0.05.

Prospective survey responses were outlined into themes related to patient perspectives. The following steps were completed: transcription, familiarization, coding and theme generation, naming and defining terms, and finalizing the dataset.^32,33^ Transcripts were independently reviewed by authors [D.F., N.A.C.], who coded phrases and clauses with identifiers. Authors [D.F, N.A.C] discussed their results, resolved discrepancies, and refined themes.

## Results

**Figure 1.**
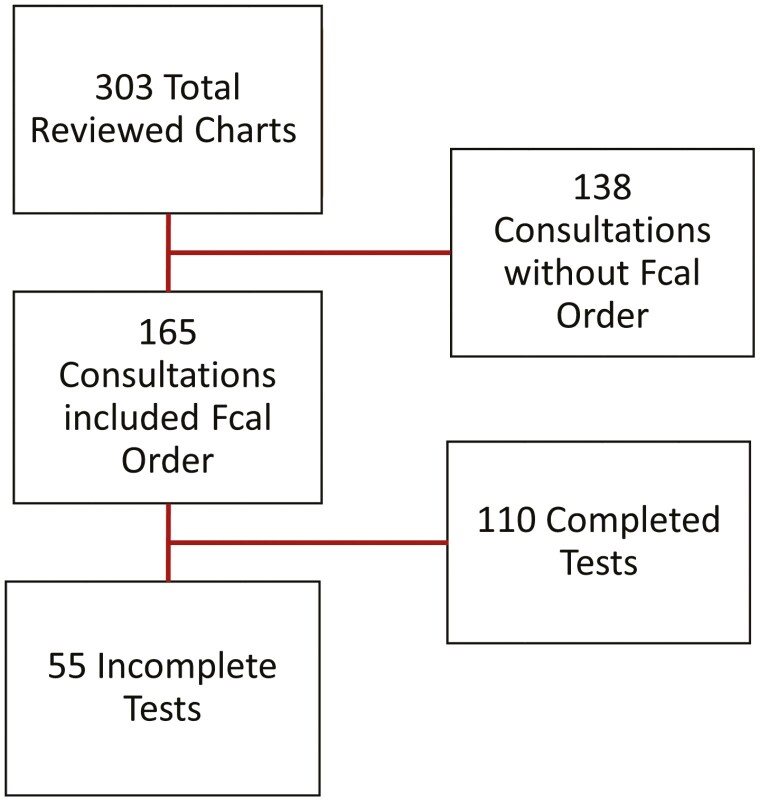
Flowchart detailing study design.

**Figure 2.**
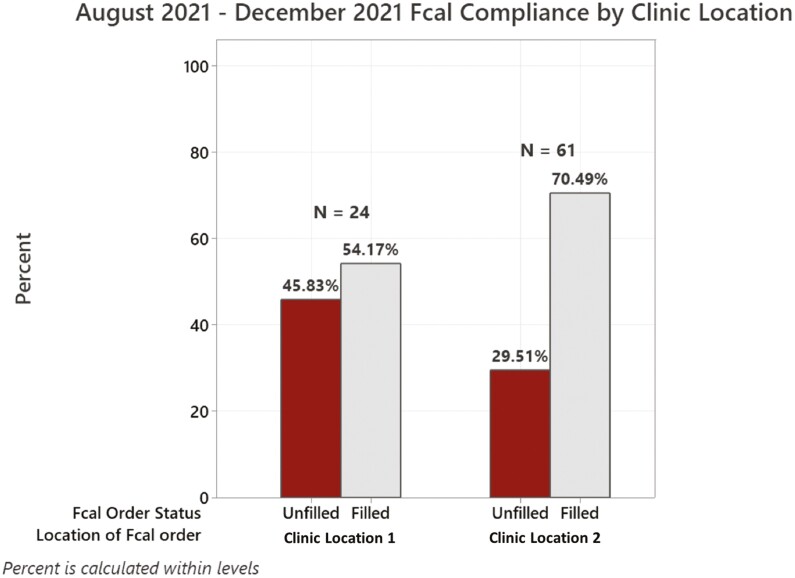
Fecal calprotectin (Fcal) compliance by clinic location.

Three hundred and three patient charts were reviewed from the [University of Chicago Medicine] IBD Center. Out of the 303, 165 (54.4%) patients’ clinic visits resulted in a subsequent Fcal order. Of all Fcal tests ordered, 55 (33.3%) were both inactive and incomplete (Figure 1). While the majority of Fcal orders were identified in between August 2021 and January 2022, 74 (44.8%) of the 165 reviewed charts contained Fcal tests in prior patient medical records. Between these dates, rates of compliance varied by clinic location (Figure 2).

### Patient Demographic and Clinical Information

Between patients with complete versus incomplete Fcal tests, median age (43.50 vs 35, *P* = .067), gender (female) (53.6% vs 49.1%, *P* = .582), race (*P* = .705), median income (98 700 vs 94 400, *P* = .418), and insurance type (*P* = .362) were similar. Compared with those with complete Fcal tests, patients with incomplete Fcal were significantly more likely to be in IBD remission status (67.8% vs 83.7%, *P* = .033), less likely to have history of prior Fcal order completion (93.2% vs 68.4%, *P* = .001), and more likely to have a test ordered to a third-party testing location (62.7% vs 85.5%, *P* = .004). Additional demographic and clinical information across cohorts is outlined in Table 1.

**Table 1.** Patient demographic and clinical information.

	Complete Fcal *N* = 110	Incomplete Fcal *N* = 55	*P*
Age (years)	43.50 (26)	35 (24)	.067
Gender (female) %	59 (53.6%)	27 (49.1%)	.582
Median income ($1000)	98.7 ± 3.40	94.4 ± 2.76	.418
Race %			
Non-Hispanic Black	4 (3.6%)	1 (1.8%)	.705
Non-Hispanic White	87 (79.1%)	46 (83.6%)	
Hispanic/Latino	1 (0.9%)	1 (1.8%)	
Non-Hispanic Asian	3 (2.7%)	3 (5.5%)	
American Indian/Alaskan Native	2 (1.8%)	—	
Native Hawaiian	2 (1.8%)	—	
Unlisted/Patient Declined	11 (10%)	4 (7.3%)	
Insurance type (%)			
Medicare	16 (14.5%)	5 (9.1%)	.362
Medicaid	3 (2.7%)	—	
Private/employment based	87 (79.1%)	49 (89.1%)	
None	4 (3.6%)	1 (<1%)	
Ulcerative colitis (%)	57 (51.8%)	28 (49.1%)	1.00
E1—ulcerative Ppoctitis	4 (8.3%)	2 (7.4%)	.619
E2—left-sided colitis	11 (22.9%)	9 (33.3%)	
E3—extensive colitis	33 (69.8%)	16 (59.3%)	
Crohn’s disease (%)	58 (52.7%)	31 (56.4%)	.741
L1—ileal	11 (23.4%)	6 (27.3%)	
L2—colonic	14 (29.8%)	6 (27.3%)	
L3—ileocolonic	22 (46.8%)	10 (45.5%)	
IBD remission (%)	59 (67.8%) *N* = 87	41 (83.7%) *N* = 49	.033
Usage of IBD-related medications (%)			
None	20 (18.3%)	6 (11.1%)	.315
1 advanced therapy (small molecule or biologic)	66 (60.6%)	39 (72.2%)	
2 or more advanced therapies (small molecule or biologic)	23 (21.1%)	9 (16.7%)	
Location of Fcal order (%)			
Clinic Location 1	46 (41.8%)	28 (50.9%)	.250
Clinic Location 2	56 (50.9%)	26 (47.3%)	
Clinic visit type (%)			
In-person	70 (63.6%)	28 (50.9%)	.132
Virtual	40 (36.4%)	27 (49.1%)	
History of fulfilled Fcal orders (%)	69 (93.2%) *N* = 74	26 (68.4%) *N* = 38	.001
Days between completed Fcal—median (IQR)	1.00 (12)	—	—
Patient location			.082
In city	27 (24.5%)	13 (23.6%)	
In state	65 (59.1%)	29 (52.7%)	
In neighboring state	14 (12.7%)	5 (9.1%)	
In distant state	4 (3.6%)	8 (14.5%)	
Fcal testing order (%)			
[Institution]	41 (37.3%)	8 (14.5%)	.004
Third-party lab	69 (62.7%)	47 (85.5%)	

Abbreviation: IBD, inflammatory bowel disease.

### Regression Models

A multivariable logistic regression including the statistically significant variables (IBD remission status, history of Fcal completion, and Fcal testing location) in addition to age, gender, telehealth versus in-person clinic visit, and patient residence revealed that history of a prior completed Fcal test is independently associated with subsequent test completion (odds ratio [OR] = 8.3, 95% confidence interval [CI] 1.9–35.5, *P* = .004). Patients with Fcal testing ordered through a third-party tended to have lower completion rates (OR = 0.27, 95% CI 0.7–1.0, *P* = .050) (Table 2). Using the same model inputs, multivariable linear regression for days between test order and completion found that tests ordered for external locations are associated with longer periods of time to test completion (95% CI 5.2–20.6, *P* = .002) (Table 2).

**Table 2.** Regression models for predictors of fecal calprotectin compliance.

Factor	Logistic regression for test completion				Regression for longer periods to Fcal test completion			
	Estimate effect size (*B*)	Std. error	*P*	95% confidence interval	Estimate effect size (*B*)	Std. error	*P*	95% confidence interval
Age (years)	0.017	0.015	.248	0.988 to 1.048	0.148	0.104	.163	−31.309 to 7.575
Gender (female)	−0.441	0.543	.417	0.222 to 1.866	0.877	3.635	.810	0.062 to 0.357
IBD remission	−0.949	0.624	.128	0.114 to 1.316	−0.331	3.895	.933	−6.421 to 8.176
Clinic visit type	−0.523	0.528	.322	0.210 to 1.570	0.102	3.992	.980	−8.151 to 7.490
History of fecal calprotectin completion	2.116	0.741	**.004**	1.942 to 35.493	6.628	2.625	.357	−7.699 to 20.956
Fecal calprotectin testing location	−1.304	0.668	**.050**	0.073 to 1.005	12.875	3.843	**.002**	5.160 to 20.591

Bold indicates the variable to be of statistical significance

Abbreviation: IBD, inflammatory bowel disease.

### Prospective Survey

**Figure 3.**
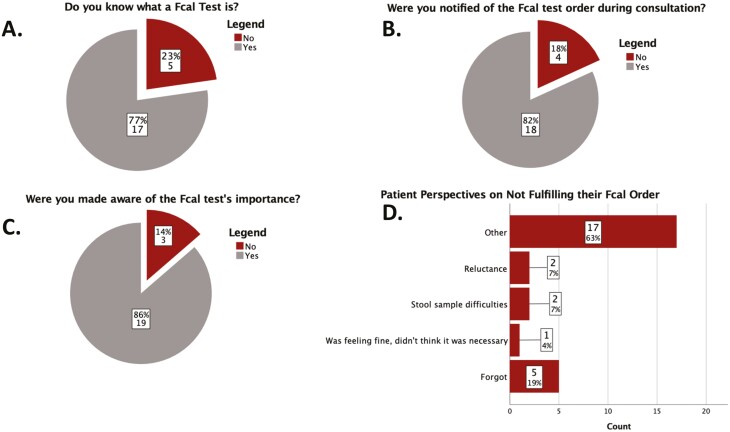
Prospective survey and patient perspectives on compliance.

Of the 55 patients with incomplete Fcal tests, 22 (40%) responded to the follow-up Fcal survey. Of the respondents, 17 (77.27%) reported knowing what a Fcal test is, 19 (86.4%) reported being aware of the Fcal test importance at the time of clinic visit, and 18 (81.8%) reported being notified of the Fcal test (Figure 3A–C). When asked for reasonings affecting their incomplete Fcal test, 5 (18.5%) reported that they forgot, 2 (7.41%) had stool collection difficulties, 2 (7.41%) were reluctant handling stool samples, and 1 (3.7%) felt fine and did not think it was necessary. Seventeen (63.0%) respondents selected “Other” and provided a short response to more closely describe their experience (Figure 3D).

### Thematic Analysis

Survey responses provided a wide array of obstacles patients faced after the initial Fcal order, ranging from COVID-19 affecting availability of testing centers and sickness preventing completion of the test, to patients describing issues in test reporting. From our thematic analysis of patient perspectives, the following themes were independently identified: pandemic-related incompletion, third-party testing issues, and patient reported test completion.

## Discussion

Compliance with IBD-monitoring tools is important to monitor response to therapy, diagnose and prevent disease flares.^34^ In turn, this may lead to control of inflammation and the prevention of disease-related complications that affect quality of life.^35^ In this study, we assessed compliance with a commonly used IBD activity marker, the Fcal test. We found that a history of Fcal completion was associated with subsequent test completion, and third-party testing locations were associated with lower completion rates. In our additional analysis and discussion with patients, we identified that many patients with records of incomplete tests held a basic understanding of the test and its importance. The effects of COVID-19 and third-party testing were other major reasons for noncompliance with order completion.

Adherence to therapeutic modifications and compliance with disease monitoring tests are regular obstacles faced by those with chronic disorders.^24,25,36^ For patients with IBD, these obstacles may be magnified by the natural course of inflammatory activity and the relapsing and remitting nature of the disease.^37^ For example, prior studies have found compliance for colorectal cancer screenings to be as low as 30%.^27,38^ In a prospective study designed to evaluate Fcal completion rates, Maréchal et al found similar Fcal completion rates to colorectal cancer screening.^39^ When assessing predictors, they found stricturing phenotype of IBD, ongoing or previous biologic treatment with infliximab or vedolizumab, and prior awareness of Fcal knowledge to all be significantly associated with noncompliance.^39^ In contrast, our present study demonstrated higher rates of compliance across clinic locations in our patient population. Clinic Location 1 had lower compliance than Clinic Location 2, likely due to adoption of telehealth visits predominantly conducted at this site past peak COVID-19 surges and increased third-party test utilization. In our initial analyses, we found that patients in remission were less likely to have completed Fcal orders. Patients’ state of remission and lack of symptoms may result in a feeling of decreased significance in their IBD management. We also found a history of no prior Fcal test completion and tests ordered through third-party locations were both associated with noncompliance. We hypothesize that patients’ prior experience may allow for greater comfortability completing orders and that patients may face additional obstacles using third-party testing centers, such as scheduling appointments or accessing testing sites, which may ultimately lead to longer periods to test completion or failure to remember. Nonetheless, compliance to IBD-monitoring tools remains a serious issue that directly affects patient health-related quality of life.

One method of increasing patient-related compliance revolves around improving education to enhance patient knowledge and emphasize health-related impacts.^40,41^ However, current research has revealed that education alone, is not an adequate means to resolve compliance issues in patients with IBD. In a systematic review aimed at determining interventions to improve medication adherence to immune-mediated inflammatory diseases, Depont et al determined that a multicomponent approach resulted in longitudinal improvements in adherence.^42^ The large proportion of patients in our study who possessed adequate health literacy regarding Fcal monitoring, yet did not complete their Fcal order underscores the importance of a multicomponent approach as an intervention. Our follow-up survey determined that a multitude of personal factors affected our patient population—ranging from forgetting, reluctance, difficulty collecting samples, etc. Clinicians should take additional time to understand patients’ positions, practices, and beliefs, and, ultimately, determine if Fcal testing is appropriate for the patient. Supplementary approaches to remind patients should be attempted such as, automated reminders or follow-up by the support team. The qualitative results of our thematic analysis point to relevant effects outside of patients’ control that providers must also reconcile.

The most common factors highlighted in patient responses were illnesses from COVID-19, issues related to third-party testing, and reports of completed testing. Patients discussed turning in samples prior to the pandemic, hesitancy related to the pandemic, or illness, which prevented completion of their Fcal order. The impact of COVID-19 has had widespread effects on chronic disease management and access to follow-up medical care. One study determined that 60% of patients with IBD had worsening symptoms from September 2020 to January 2021, likely from decreased medical care access and reduction of therapeutic monitoring.^43^ These findings necessitate additional research determining the COVID-19 pandemic impacts on provider utilization and patient compliance with Fcal testing. We also identified issues regarding third-party testing and subsequently, reports of test completion from patient responses. Several patients described issues with scheduling and coordination, importing results to our clinics, or general issues with testing. We hypothesize that issues with importing or transferring test results may have led to the discrepancy between patient records and their reporting of Fcal completion. The importance of this is compounded by the decrease in compliance associated with third-party testing locations. One future direction to improve ease and practicality for IBD monitoring includes self-monitoring through at-home Fcal tests.^44^ While self-monitoring options present as a method to circumnavigate difficulties associated with third-party testing, more research should be conducted to determine discrepancies in patient test records with third-party testing options.

From our findings, we have outlined practical guidelines that healthcare teams may follow to improve patient monitoring and maximize institutional rates of Fcal completion (Table 3).

**Table 3.** Applicable recommendations for fecal calprotectin ordering.

Related factors for Fcal incompletion	Recommendations for improvement
1. History of Fcal incompletion	Providers should spend more time understanding barriers patient faces in completing testing.
	Patient history of other IBD-monitoring tests should be evaluated to determine any potential trends.
	If patients lack previous experience with Fcal testing, providers should explain rationale and describe importance of the test.
	Care teams can provide timely follow-up after a clinic visit to determine if there are any issues and ensure completion.
2. Third-party testing location	If timely modifications to therapy are required, providers should note potential delays in test completion from scheduling, forgetfulness, etc.
	Patients should be followed-up 1 week after the initial order placement to remind them to complete the test and determine if they face any obstacles.
	Providers should be willing to help coordinate appointment scheduling if conflicts arise.
	Providers should ensure results are imported back into patient charts in a timely manner.
3. Pandemic-related effects	Providers should address patient’s potential fears about exposure to COVID-19 at healthcare facilities and testing sites.
	If patient contracts COVID-19, follow-up with patient should be conducted to ensure completion of Fcal test.

Abbreviation: IBD, inflammatory bowel disease.

While this study leveraged both retrospective and prospective design components, our study has several limitations. First, this study was conducted at a single center, and the patients were all seen by a single clinician. Another limitation includes the time between the identified Fcal incompletion and the prospective survey. While our review included all encounters between January 2018 and January 2022, patients may have had subsequent visits. Furthermore, there may have been patient recall bias on testing. Finally, the number of patients who completed the secondary prospective survey was small, and limited the strength of the thematic analysis. Notwithstanding these limitations, the study has numerous strengths, including quantitatively determining predictors and qualitatively revealing insight from patient perspectives. Moreover, our study cohort is diverse, including patients from both the hospital campus and community-based off-site clinics and with a variety of income levels and insurance providers, making our findings generalizable.

## Conclusion

In conclusion, IBD management requires compliance with noninvasive monitoring tools such as Fcal. In this single center analysis with Fcal testing in patients with IBD, we found that a history of incomplete testing is associated with subsequent noncompliance. Patients highlighted difficulties related to COVID-19 and third-party testing locations. We provided practical guidelines based on the findings of this study. However, additional studies should be completed to outline the effects of COVID-19 on Fcal compliance and determine the potential discrepancies amongst third-party testing.

## Supplementary Material

otac042_suppl_Supplementary_Data_S1

otac042_suppl_Supplementary_Data_S2

## Data Availability

Data collected for this study contain sensitive information on patients that make up the cohort and may be made available upon request from the corresponding author.
