# Supplementary material for: Factors Associated With Fecal Calprotectin Sample Collection Compliance: An IBD Center Quality Improvement Project
Source: Crohns Colitis 360. 2022 Dec 3;4(4):otac042. doi: 10.1093/crocol/otac042 (PMC9802166; doi:10.1093/crocol/otac042)
Supplement: otac042_suppl_Supplementary_Data_S1 [file otac042_suppl_supplementary_data_s1.pdf]

# Fcal Database

MRN ______________________

## Patient Demographic Information

Age ______________________

Patient sex
- ◯ Male
- ◯ Female

Patient Race/Ethnicity
- ☐ Non-Hispanic Black
- ☐ Non-Hispanic White
- ☐ Hispanic/Latinx
- ☐ Non-Hispanic Asian
- ☐ American Indian/ Alaskan Native
- ☐ Native Hawaiian/ Pacific Islander
- ☐ Not Listed

Please Specify ______________________

Highest Level of Education
- ◯ Some High School
- ◯ High School Degree/ GED
- ◯ Associate's Degree
- ◯ Bachelor's Degree
- ◯ Master's Degree
- ◯ Doctoral Degree

Zipcode ______________________

## Medical History

Has the patient been diagnosed with IBD?
- ◯ Yes
- ◯ No

Date of IBD diagnosis? ______________________

Which was the patient diagnosed with?
- ☐ Ulcerative Colites (UC)
- ☐ Crohn's Disease (CD)

What type of Ulcerative Colitis was the patient diagnosed with?
- ☐ Ulcerative proctitis - anus (rectum)
- ☐ Left-sided colitis - rectum up through the sigmoid to splenic flexure
- ☐ Extensive Colitis

What type of Crohn's Disease was the patient diagnosed with?
- ☐ L1 - ileal
- ☐ L2 - Colonic
- ☐ L3 - Ileocolonic
- ☐ L4 - Proximal involvement
- ☐ P - (modifier) perianal disease

| | |
|---|---|
| Harvey Bradshaw Index (Crohn's disease) | __________________________________ |
| Simple Clinical Colitis Activity Index (Ulcerative Colitis) | __________________________________ |
| History of the following GI-related diseases/issues | ☐ Irritable Bowel Syndrome<br>☐ Clostridium Difficile<br>☐ Dyspepsia<br>☐ Gastroesophageal Reflux Disease (GERD)<br>☐ Other |
| Please specify | __________________________________ |
| Is the patient on any of the following medications for GI-related issues? | ☐ Infliximab (Remicade/Inflectra)<br>☐ Adalimumab (Humira)<br>☐ Golimumab (Simponi)<br>☐ Certolizumab (Cimzia)<br>☐ Vedolizumab (Evntyvio)<br>☐ Ustekinumab (Stelara)<br>☐ Tofacitinib (Xeljanz)<br>☐ Ozanimod (Zeposia)<br>☐ Ciproflaxacin (Cipro)<br>☐ Metronidazole (Flagyl)<br>☐ Azathioprine (Azasan, Imuran)<br>☐ Mercaptopurine (Purinethol, Purixan)<br>☐ Methotrexate (Trexall)<br>☐ Mesalamine (Asacol, HD, Delzicol)<br>☐ Balsalazide (Colazal)<br>☐ Olsalazine (Dipentum)<br>☐ Prednisone<br>☐ None<br>☐ Other |
| Please specify | __________________________________ |
| History of the following medical issues | ☐ Hypertension<br>☐ Diabetes Mellitus<br>☐ Hyperlipidemia<br>☐ Cancer<br>☐ Coronary Heart Disease<br>☐ Congestive Heart Failure<br>☐ Degenerative Joint Disease (DJD)<br>☐ COPD<br>☐ None<br>☐ Other |
| Please specify | __________________________________ |
| History of previous surgeries | ◯ Yes<br>◯ No |
| Surgery Type | __________________________________ |

| | |
|---|---|
| History of previous hospitalizations | ◯ Yes<br>◯ No |

## Clinical Encounter Information

| | |
|---|---|
| Date of Fcal order | ______________________ |
| Location of Fcal order | ◯ River East<br>◯ Hinsdale<br>◯ Hyde Park<br>◯ NorthShore |
| Was the consultation in-person or virtual? | ◯ In-person<br>◯ Virtual |
| Patient from Chicago or out-of-state? | ◯ Chicago<br>◯ Illinois<br>◯ Neighboring State<br>◯ Distant State |
| Where was the testing location ordered for? | ◯ University of Chicago Med<br>◯ Third-party lab<br>◯ Other |
| Was the Fcal order completed? | ◯ Yes<br>◯ No |
| Date of Fcal Order Filled | ______________________ |
| Has the patient had previous Fcal testing orders? | ◯ Yes<br>◯ No |
| Has the patient filled previous Fcal orders? | ◯ Yes<br>◯ No |
| What type of insurance does the patient have? | ◯ Medicare<br>◯ Medicaid<br>◯ Employment-based<br>◯ Private Insurance<br>◯ None<br>◯ Other |
