# Supplementary material for: Factors Associated With Fecal Calprotectin Sample Collection Compliance: An IBD Center Quality Improvement Project
Source: Crohns Colitis 360. 2022 Dec 3;4(4):otac042. doi: 10.1093/crocol/otac042 (PMC9802166; doi:10.1093/crocol/otac042)
Supplement: otac042_suppl_Supplementary_Data_S2 [file otac042_suppl_supplementary_data_s2.pdf]

University of Chicago IBD Center Fecal Calprotectin Survey

Hello, this is Dr. Rubin’s team from the University of Chicago Inflammatory Bowel Disease (IBD) Center. We noted an incomplete Fecal Calprotectin test in our records from 2018-2022. Please help us better understand how to improve upon services at the University of Chicago IBD Center by answering the following questions.

Thank you!

---

1. Do you know what a Fecal Calprotectin test is?
   - ◯ Yes
   - ◯ No

1a. Fecal calprotectin is a protein used to assess gut inflammation for patients. Calprotectin testing involves collecting patient stool samples in containers and determining the levels of the protein in the stool. Here at the University of Chicago IBD Center, fecal calprotectin testing is a very important way for us to determine therapy options and monitor for IBD relapse without conducting a colonoscopy.

---

2. Were you notified that a Fecal Calprotectin test was ordered?
   - ◯ Yes
   - ◯ No

---

3. Were you made aware of the importance of Fecal Calprotectin testing during your consultation?
   - ◯ Yes
   - ◯ No

---

4. Which reasons best describe why you might have been unable to complete the Fecal Calprotectin test? Please select all that apply:
   - ☐ Forgot
   - ☐ Didn't understand its importance
   - ☐ Was feeling fine and didn't think it was necessary
   - ☐ Was feeling ill and didn't think it would add anything to my management
   - ☐ Stool collection difficulties (i.e. constipation, diarrhea, etc.)
   - ☐ Reluctance in handling stool samples
   - ☐ Confusion around testing location
   - ☐ Other:

4a. If you selected "other", please explain:

____
